# Bayesian localization microscopy based on intensity distribution of fluorophores

**DOI:** 10.1007/s13238-015-0133-9

**Published:** 2015-02-12

**Authors:** Fan Xu, Mingshu Zhang, Zhiyong Liu, Pingyong Xu, Fa Zhang

**Affiliations:** 1 Key Lab of Intelligent Information Processing, Institute of Computing Technology, Chinese Academy of Sciences, Beijing, 100190 China; 2 University of Chinese Academy of Sciences, Beijing, 100049 China; 3 Laboratory of Non Coding RNA, Institute of Biophysics, Chinese Academy of Sciences, Beijing, 100101 China

**Keywords:** super-resolution, fluorescence image, 3B, intensity distribution

## Abstract

Super-resolution microscopy techniques have overcome the limit of optical diffraction. Recently, the Bayesian analysis of Bleaching and Blinking data (3B) method has emerged as an important tool to obtain super-resolution fluorescence images. 3B uses the change in information caused by adding or removing fluorophores in the cell to fit the data. When adding a new fluorophore, 3B selects a random initial position, optimizes this position and then determines its reliability. However, the fluorophores are not evenly distributed in the entire image region, and the fluorescence intensity at a given position positively correlates with the probability of observing a fluorophore at this position. In this paper, we present a Bayesian analysis of Bleaching and Blinking microscopy method based on fluorescence intensity distribution (FID3B). We utilize the intensity distribution to select more reliable positions as the initial positions of fluorophores. This approach can improve the reconstruction results and significantly reduce the computational time. We validate the performance of our method using both simulated data and experimental data from cellular structures. The results confirm the effectiveness of our method.

**Electronic supplementary material:**

The online version of this article (doi:10.1007/s13238-015-0133-9) contains supplementary material, which is available to authorized users.

## Introduction

Fluorescence microscopy, which enables the observation of living cell structures, organelles and even small molecules, plays an indispensable role in life science. However, the spatial resolution of conventional light microscopy is restricted to approximately half the emission wavelength due to optical diffraction (Hell, 2007). To overcome this limitation, several super-resolution fluorescence microscopy techniques based on single-molecule localization have been developed in recent years, such as stochastic optical reconstruction microscopy (STORM) (Rust et al., 2006), photo-activated localization microscopy (PALM) (Betzig et al., 2006) and fluorescence PALM (fPALM) (Hess et al., 2006). These techniques utilize the on-off switching of fluorescent probes to ensure that each active fluorophore is isolated beyond the range of diffraction-limitation and ultimately build a high-resolution image from the precise and accurate positions of many single fluorophores (Deschout et al., 2014). However, single-molecule localization techniques require that the density of fluorophores in each frame remains sufficiently low to prevent individual fluorophores from overlapping, which leads to long imaging times and increases the damage to live samples (Lippincott-Schwartz and Manley, 2008). Thus, low temporal resolutions limit the application of super-resolution microscopy techniques in live-cell imaging. Although several methods have been developed based on simultaneous fitting with multiple fluorophores to deal with relatively dense fluorescent data (DAOSTORM, 2011; Huang et al., 2011; Quan et al., 2011), the localization accuracy of fluorophores dramatically decreases as the density of emitters increases.

Recently, a localization microscopy analysis method named the Bayesian analysis of bleaching and blinking (3B) method was developed to address the high-density fluorophore data extracted from live cells with standard fluorescent proteins (Cox et al., 2012). In the analysis of 3B, the entire image sequences are modeled as a set of blinking and bleaching fluorophores, and the properties of blinking and bleaching are utilized by hybridizing two hidden Markov model inference methods to improve the obtained accuracy of fluorophore positions.

During the analysis, 3B uses the changed information by adding or removing fluorophores in the cell to adjust the model and further fit the data. When adding a new fluorophore, the initial selected position is random, and the position prior is assumed to be uniform in the optimization iteration of 3B. 3B optimizes this random position to determine whether to keep this position as a true single molecule in the model. In fact, the fluorophores are not evenly distributed in the entire image region, and the fluorescence intensity positively correlates with the probability of observing a fluorophore at this position. If a presumed initial position is far away from the real biological structure, re-optimizing this position is a waste of time and leads to inaccurate results. Thus, assuming that the position prior of fluorophores is uniform in 3B is not appropriate.

In this article, we propose a Bayesian analysis of Bleaching and Blinking microscopy method based on the fluorescence intensity distribution (FID3B) to improve the reconstruction results and accelerate computation. The key techniques include two aspects: an intensity distribution calculation to obtain the probability distribution of fluorophores and a modified model to add a procedure that selects the initial positions of fluorophores based on the intensity distribution. In 3B, each fluorophore transitions between the emitting state, non-emitting state and bleached state according to a Markov process, and each transition is associated with a transition probability. The transition probabilities, together with the fluorophore’s previous state, are used to determine the state of a fluorophore at a certain time. The fluorescence intensity represents the probability of observing a fluorophore at this position. We calculated the intensity distribution of fluorophores at each pixel by combining transition probabilities and fluorescence intensity. Instead of choosing randomly, we then selected a position with more confidence for each newly added fluorophore according to previous knowledge. As a consequence, the results of our method are much more consistent with the real structure, and the computational time can be significantly reduced. Both simulated data and real cellular structures were tested to validate the performance of our method. The results provide convincing evidence of the effectiveness of our method.

## Results

### Experimental validation using simulated dataset

**Figure 1:**
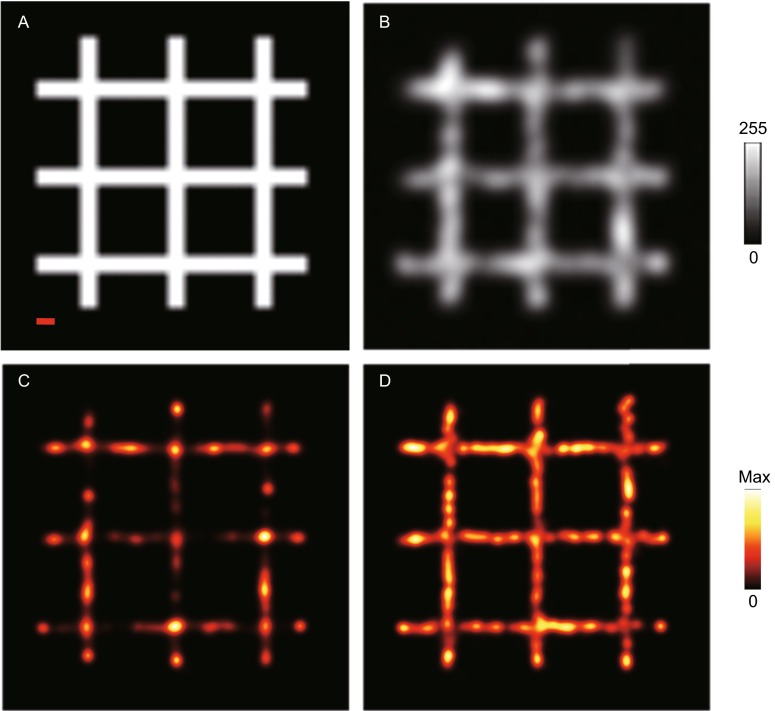
**Comparison of the reconstruction results of 3B and FID3B for the grid dataset**. (A) The original image of the grid data. (B) Superimposed fluorescence data from 200 frames representing the diffraction-limited image. (C) Reconstruction result of 3B (320 iterations). (D) Reconstruction result of FID3B (320 iterations). (Scale bar: 200 nm). Note that the result of FID3B is much more similar to the original image, while the result of 3B shows many discontinuous areas

**Figure 2:**
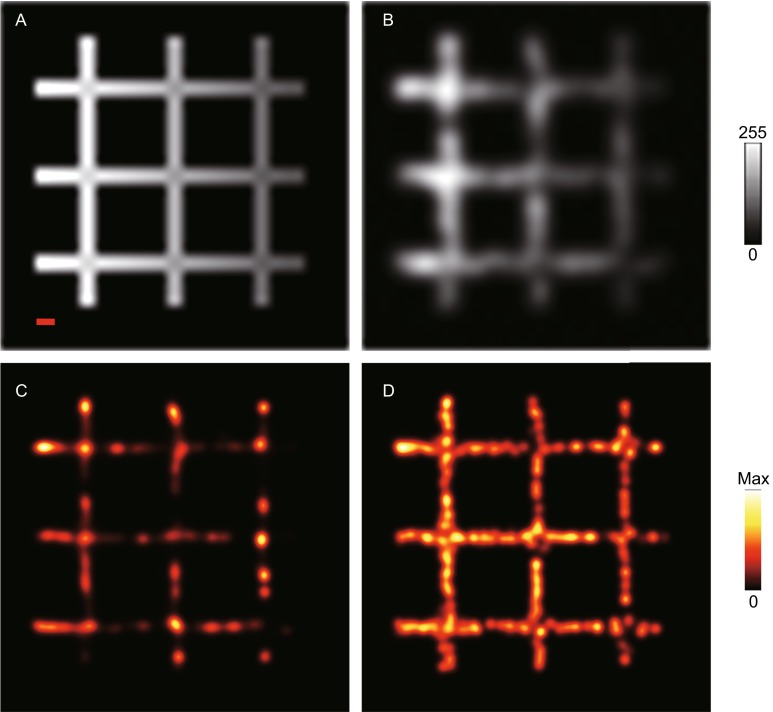
**Comparison of the reconstruction results of 3B and FID3B for the gradient grid dataset**. (A) The original image of the gradient grid data (Brightness gradually decays from left to right). (B) Superimposed fluorescence data from 200 frames representing the diffraction-limited image. (C) Reconstruction result of 3B (240 iterations). (D) Reconstruction result of FID3B (240 iterations). (Scale bar: 200 nm). Note that the result of FID3B exhibits the same brightness gradient as the original image and thus is much more similar to the real image

We present two different simulated datasets to demonstrate the performance of FID3B (Figs. 1 and 2). One is a grid structure composed of 6 intersecting lines with evenly distributed brightness, named the grid dataset (Fig. 1A); the other is also a grid structure composed of 6 intersecting lines, but with gradually decaying brightness from left to right, named the gradient grid dataset (Fig. 2A). Both datasets consist of 200 image frames, and the size of each frame is 40 × 40 pixels (1 pixel = 100 nm). Here, the full width at half maximum (FWHM) of the optical point spread function (PSF) was set to 240 nm.

Figure 1 depicts the reconstruction results of 3B and FID3B with the grid dataset. According to the characteristics of the state transition of fluorophore in 3B, we generated the grid dataset of 200 frames from the original image (Fig. 1A). The fluorescence density of each frame was very high, and the fluorescent molecules were overlapped. The superimposed fluorescence data from 200 frames are shown in Fig. 1B. Both 3B and FID3B were run with a fixed number of iterations, 320, and the reconstruction results are shown in Fig. 1C and 1D. The brightness of the image that resulted from 3B is relatively low, and the lines in the grid are discontinuous in many areas. In contrast, the reconstruction result of FID3B retains more details and thus is much more similar to the original image.

To validate the ability to process images with unevenly distributed brightness, the reconstruction results of 3B and FID3B with the gradient grid dataset are shown in Fig. 2. As for the previous grid dataset, the gradient grid dataset of 200 frames was generated from the original image (Fig. 2A). The brightness of the original image gradually decays from left to right. The superimposed fluorescence data from 200 frames are shown in Fig. 2B. Both 3B and FID3B were run with a fixed number of iterations, 240, and the reconstruction results are shown in Fig. 2C and 2D. In contrast to the reconstruction result of 3B, the reconstruction result of FID3B reveals more details. Moreover, the reconstruction result of FID3B exhibits the same brightness gradient as the original image and thus is much more similar to the real image.

To validate the capability of FID3B in resolving fine structures, we generated simulated images with concentric rings structures, where the distance between two neighboring rings is 200 nm (Fig. S1). Although it is difficult to see the concentric rings structures from the superimposed fluorescence data, the reconstruction results of both 3B and FID3B are consistent with the original image. Moreover, the reconstruction result of FID3B has better continuity.

### Quantitative analysis of image reconstruction quality

In previous studies, quantitative measurements have been used to evaluate the performance and quality of localization microscopy algorithms (Ram et al., 2006; Small, 2009; Huang et al., 2011; Wolter et al., 2011). These measurements assess the detection and localization accuracy of single or multi emitters in each frame. Because 3B analyzes the entire image sequences to obtain a probability map of the positions of fluorophores, which does not reflect the real localization of fluorophores (Lidke, 2012), we sought to measure the quality of the overall reconstructed image instead of the location of single or multi emitters. The similarity between the original image and reconstructed image is a good indicator of the reconstruction quality. In this work, the structural similarity (SSIM) (Wang et al., 2004), which is widely used in the digital image process field, was used to measure the similarity.

SSIM assesses the visual impact of three aspects of an image: luminance, contrast and structure, which are also the main concern of 3B and FID3B. A large SSIM value represents high similarity. The measurement between the original image *x* and reconstructed image *y* is defined as follows:

1 $${\rm{SSIM}}({\text{x,y}}) = \frac{{(2\mu_{x} \mu_{y} + C_{1} )(2\sigma_{xy} + C_{2} )}}{{(\mu_{x}^{2} + \mu_{y}^{2} + C_{1} )(\sigma_{x}^{2} + \sigma_{y}^{2} + C_{2} )}}$$

where *μ*_*x*_ and *μ*_*y*_ are the mean values of *x* and *y*, $\sigma_{x}^{2}$ and $\sigma_{y}^{2}$ are the variances of *x* and *y*, *σ*_*xy*_ is the covariance of *x* and *y*, and *C*_1_ and *C*_2_ are two variables to stabilize a division with a weak denominator.

**Figure 3:**
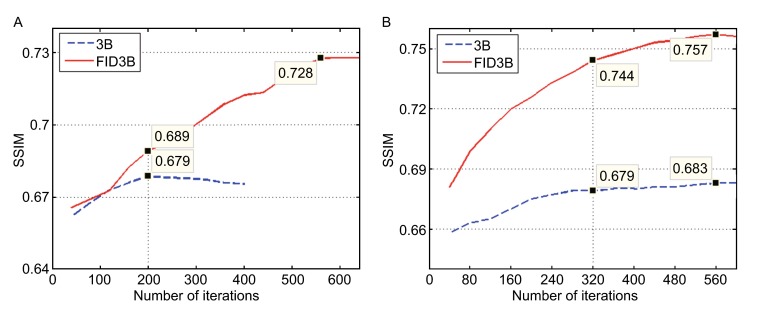
**Quantitative comparison of the reconstruction results of the grid and gradient grid datasets with 3B and FID3B**. (A) Measuring image quality of the grid dataset with SSIM. (B) Measuring image quality of the gradient grid dataset with SSIM

Figure 3 illustrates the quantitative comparison between 3B and FID3B with the grid dataset and the gradient grid dataset. The experimental output data of both 3B and FID3B were recorded at an interval of 40 iterations. For every intermediate output, their similarity with the original image was measured by SSIM. The similarity curve shown in Fig. 3 indicates that FID3B is superior to 3B. The grid dataset and gradient grid dataset mentioned above were used to evaluate the reconstructed image. For the grid dataset (Fig. 3A), 3B no longer improved the reconstructed image quality after 200 iterations, but FID3B continued to improve the reconstructed image quality after 200 iterations. When convergence was reached, the similarity value of 3B was 0.679, which was 0.728 of FID3B. For the gradient grid dataset (Fig. 3B), the image quality of 3B essentially stopped improving after 320 iterations. When convergence was reached, the similarity value of 3B was 0.683, and the similarity value of FID3B was 0.757. The similarity value of FID3B is clearly higher than that of 3B for the same number of iterations.

In order to demonstrate the acceleration effect of our method, experiments of both 3B and FID3B with the grid dataset mentioned above were carried out on a platform with an Intel E7500 (2.93 GHz) CPU. We used SSIM to measure the similarity between the original and reconstructed images and recorded the computational time of 3B and FID3B. We ran both 3B and FID3B in parallel for 2 days. 3B required nearly 38.5 h to converge to its best similarity value (0.679), while FID3B required only 25.2 h to achieve the same similarity value and continued to improve the result to a large extent.

### Evaluating the performance by experimental data of cellular structure

**Figure 4:**
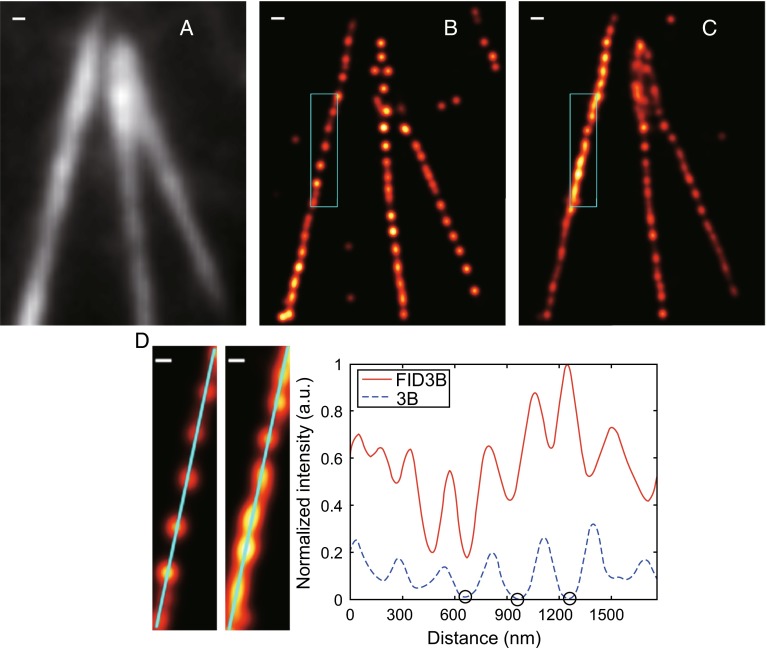
**Comparison of the reconstruction results of the two methods based on real experimental data**. (A) Superimposed fluorescence data from 200 frames data showing diffraction-limited resolution. (B) Reconstruction result of 3B (160 iterations). (C) Reconstruction result of FID3B (160 iterations). (D) Magnification of solid box in B and C, showing the distribution of fluorescence intensity along the solid line. The black cycles show some points of which the density is nearly down to 0. The scale bars are 200 nm (A–C) and 100 nm (D). Note that the image continuity obtained with FID3B is better and much more consistent with the real structure

To evaluate the performance of our method in biological samples, COS7 cells expressing mEos3.2-labeled Lifeact, an actin binding peptide, were illuminated at 488 nm and imaged with the total internal reflection fluorescence microscope (Fig. 4). The experimental data consisted of 200 image frames, and the size of each frame was 38 × 50 pixels (1 pixel = 100 nm). The superimposed fluorescence data from 200 frames show the diffraction-limited image (Fig. 4A). Two methods, 3B and FID3B, were used to process the experimental data and reconstruct the final super-resolution images (Fig. 4B and 4C). Both methods were run with 160 iterations. The reconstruction result of 3B shows many inconsecutive point structures, while the reconstruction result of FID3B shows improved image continuity and is much more consistent with the real structure. Furthermore, some missing structures marked by solid box in Fig. 4B are clearly visualized in Fig. 4C. The magnification of the solid boxes is shown in Fig. 4D. We calculated the distribution of fluorescence intensity along the solid line. In this distribution of fluorescence intensity, FID3B is denser than 3B at all points of the curve. At some points, the density of 3B is almost 0 (as shown by the black cycles in Fig. 4D). These points correspond to inconsecutive structures in the magnified area.

## Discussion

We propose a Bayesian analysis of Bleaching and Blinking microscopy method based on fluorescence intensity distribution (FID3B). Our method introduces statistical analysis to estimate the distribution probability of fluorescently labeled biological structures in images, and these data are utilized to further guide the selection of the initial positions of fluorophores. We validated our method with both simulated fluorescence data and experimental data from cellular structures. These experiments show that our method better addresses both images with even and uneven brightness. Compared with 3B, our method selects a better starting position when adding a new fluorophore and involves more positions in computation at the same number of iterations. As a result, the reconstruction results can be improved, and the computational time can be significantly reduced.

We explain how our method affects the reconstruction results based on two aspects: the selection of the initial positions of fluorophores and the number of retained and discarded positions of fluorophores.

### Comparison of the distribution of initial positions of fluorophores

**Figure 5:**
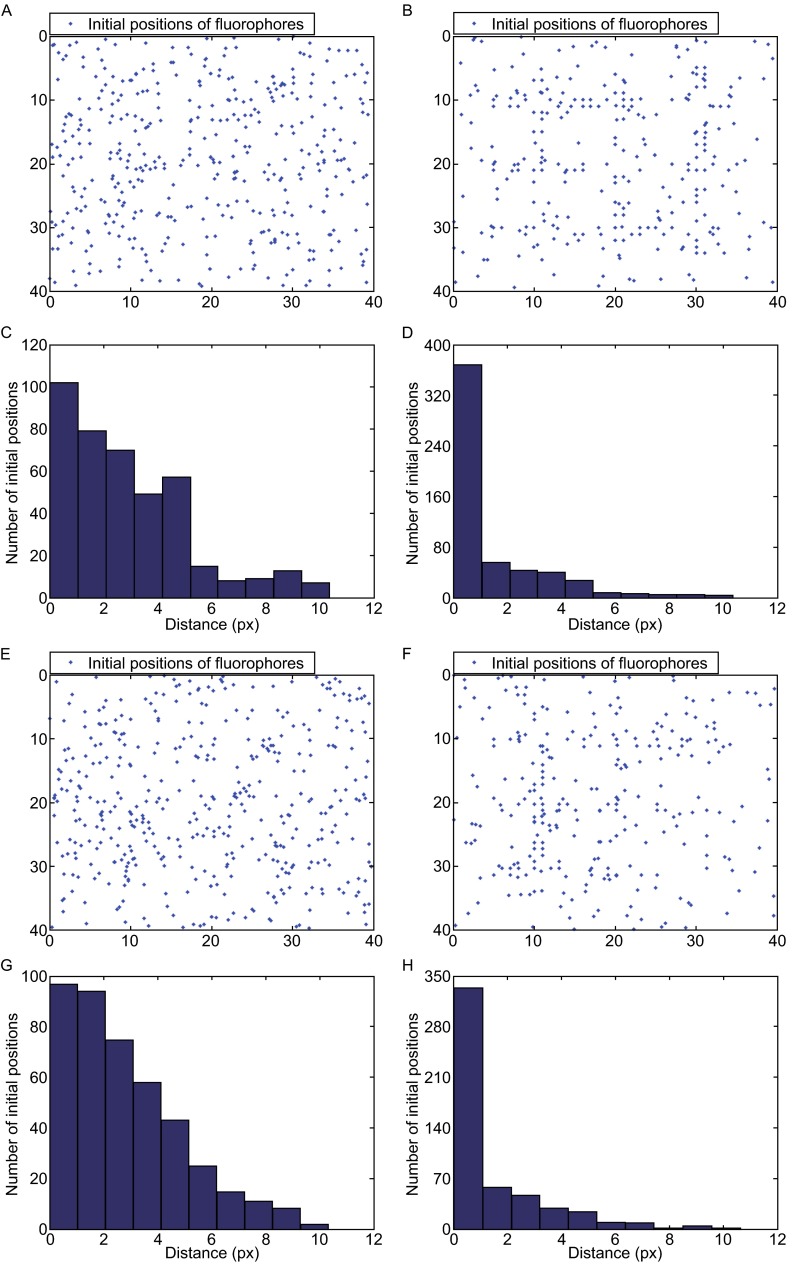
**Comparison of the distribution of initial positions for the grid dataset (A–D) and the gradient grid dataset (E–H)**. (A) Distribution of initial positions using 3B. (B) Distribution of initial positions using FID3B. (C) Distances from initial positions to image contour in 3B. (D) Distances from initial positions to image contour in FID3B. (E–H) are interpreted as the same as (A–D)

To illustrate the selection of the initial positions of fluorophores, we compared the distribution of these generated positions using 3B and FID3B. Both 3B and FID3B experiments were carried out with the grid dataset and gradient grid dataset mentioned above for the same 320 iterations. We recorded the initial positions selected by each method and calculated the distance from each initial position to the nearest real molecule in the structure in each dataset. For the grid dataset (Fig. 5A–D), the number of initial positions of fluorophores was 407 for 3B and 562 for FID3B. Comparing the geometry distributions of initial positions in Fig. 5A and 5B to the corresponding original images mentioned above indicated that the positions of the selected initial fluorophores of FID3B are much closer to the real structures. The histograms of the distances from initial positions to the nearest structure provide solid support for our findings (Fig. 5C and 5D). The number of selected initial positions with distances less than 1 is 100 in 3B, which consisted of only 24.6% of total initial positions. In contrast, 367 initial positions with distances less than 1 are observed in FID3B, comprising up to 65.3% of total initial positions. For the gradient grid dataset (Fig. 5E–H), the initial positions of FID3B are also much closer to the structure of the original image (Fig. 5E and 5F). The results from 3B and FID3B include 427 and 518 initial positions, respectively. The histograms of distances are shown in Fig. 5G and 5H. The number of selected positions with distances less than 1 in FID3B is 331 (63.9% in total), while in 3B this number is 90 (21.1%). The above comparison confirms the advantage of FID3B in selecting initial positions.

### Comparison of the number of retained and discarded positions of fluorophores

**Figure 6:**
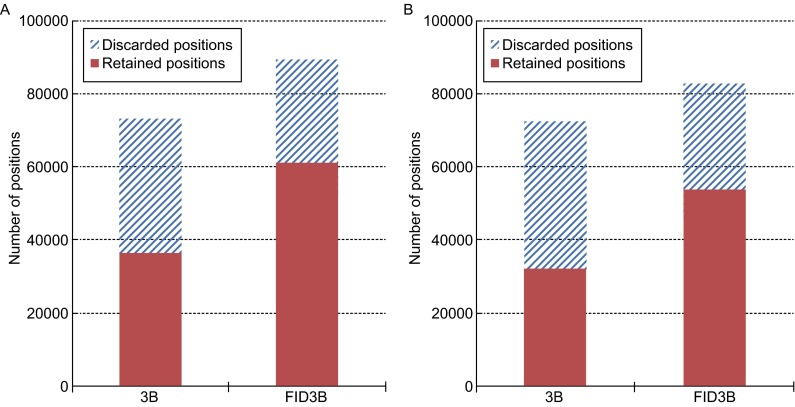
**Comparison of the number of retained and discarded positions for two datasets**. (A) Grid dataset (B) Gradient grid dataset

3B uses the change in information caused by adding or removing fluorophores in the cell to fit the data. When adding a new fluorophore, 3B selects an initial position, optimizes this position, and then determines whether it is a reliable position of a fluorophore. If the position is reliable, it will be retained and referred to as a retained position; otherwise, it will be discarded and referred to as a discarded position. We ran both 3B and FID3B with the two datasets mentioned above for the same 320 iterations and recorded the numbers of retained and discarded positions (Fig. 6). For the grid dataset (Fig. 6A), 3B yielded 36,756 discarded positions and 36,311 retained positions, resulting in a total of 73,067 positions with a discard rate of 50.3%. In contrast, FID3B yielded 28,036 discarded positions and 61,248 retained positions, resulting in 89,284 positions in total with a much lower discard rate of 31.4%. For the gradient grid dataset (Fig. 6B), 3B yielded 40,792 discarded positions and 31,938 retained positions, resulting in a total of 72,730 positions with a discard rate of 56.1%. In contrast, FID3B yielded 28,736 discarded positions and 53,872 retained positions, resulting in 82,608 positions in total with a much lower discard rate of 34.8%. Fig. 6 shows that FID3B clearly involves more positions in computation for the same number of iterations, as a result, the reconstruction result of FID3B is much closer to the real structure than that of 3B.

## Materials and Methods

### Plasmids construction

To express Lifeact-mEos3.2 in mammalian cells, mEos3.2 containing *Bam*HI and *Not*I sites was first PCR-amplified and inserted into the pmEos3.2-N1 (Clonetech) plasmid to replace EGFP. The Lifeact sequence was then cloned into pmEos3.2-N1 with *Eco*RI and *Bam*HI. The synthetic DNA primers used for cloning were purchased from Invitrogen. All plasmids were sequenced (The Beijing Genomics Institute) before further analysis.

### Cell culture, transfection and fixation

COS-7 cells were cultured in DMEM complete medium (Gibco) supplemented with 10% fetal bovine serum and maintained at 37°C in a humidified incubator (Thermo). They were then transiently transfected using Lipofectamine^TM^ 2000 (Invitrogen) in accordance with the manufacturer’s protocol when they reached 80% confluence. Before fixation, the cells were grown in DMEM complete medium (Gibco) for 24 h. The cells were then sub-cultured on coverslips (Fisher Scientific) for another 24 h and fixed with 3% (*w*/*v*) paraformaldehyde and 0.5% glutaraldehyde in PBS for 15 min at 37°C, washed 3 times with filtered PBS and stored in PBS until imaging.

### Optical setup and imaging

The 3B imaging of Lifeact-mEos3.2 was performed as previously described (Cox et al., 2012). We used an Olympus IX71 inverted microscope equipped with a 100 × 1.45 numerical aperture (NA) oil objective (Olympus PLAN APO). An internal 1.6× magnification was used to yield a pixel size of 100 nm. An acousto-optic tunable filter (AA Optoelectronic) was used to control the 488-nm laser (Sapphire). The fluorescence signals were acquired using an electron-multiplying charge-coupled device (EMCCD) camera (Andor iXon DU-897 BV). For 3B imaging, Lifeact-mEos3.2 constructs were imaged by a 488-nm laser with 50 ms integration times. The 3B datasets consisted of 200 frames and were corrected for drift.

### Simulated dataset generation

We generated two different simulated datasets with overlapping fluorophores in each frame. One was the grid dataset, whose brightness was uniform in the grid region; the other was the gradient grid dataset, whose brightness gradually decayed from left to right. Both datasets consisted of 200 image frames, and the size of each frame was 40 × 40 pixels (1 pixel = 100 nm). In all of our simulations, the optical point spread function (PSF) was represented as a 2D Gaussian shape with a width parameter of 100 nm. In 3B, the entire dataset was generated from large numbers of fluorophores that had blinking and bleaching properties. All of these fluorophores were modeled after a Factorial Hidden Markov Model (FHMM) (Ghahramani and Jordan, 1997), each of which was modeled after a Hidden Markov Model (HMM) (MacKay, 2003) and had three possible states: emitting (light), not emitting and bleached. The fluorophore can transition between the emitting and not emitting state as well as from the not emitting to the bleach state. Once it has transferred to the bleach state, the fluorophore can no longer transfer to the other states. We assumed that the states of all fluorophores were statistically independent. These characteristics of state transition proposed in 3B were used to generate simulated datasets. In the first frame of the simulation, half of the fluorophores were in the emitting state and half were in the not emitting state. Subsequently, we created an image for each frame that consisted of fluorophores whose states were randomly decided by a state transition diagram. The stack of image frames was degraded by both shot (Poisson) noise and read out (Gaussian) noise.

### Bayesian analysis of bleaching and blinking (3B) method

3B utilizes a Bayesian model to generate fluorescence images with a spatial resolution approaching 50 nanometers. It can handle the high-density fluorophore image data extracted from live cells with standard fluorescent proteins. In this Bayesian technique, 3B models the entire image sequences as a set of blinking and bleaching fluorophores and generates a probability map of positions using a maximum a posteriori (MAP) calculation.

The complete procedures for 3B are summarized as follows:

- ***Step 1.*** Select the initial spot positions for a model.
- ***Step 2.*** Optimize the entire model: re-optimizing each fluorophore in turn to obtain a new position in the model.
- ***Step 3.*** Model selection: incrementally adjusting the model to fit the data, one fluorophore at a time. 3B either adds a new fluorophore at a random position or selects a fluorophore in the model for removal.

Repeating *Step 2* and *Step 3* generates a super-resolution fluorescence image. The algorithm is terminated when the adjacent reconstructed images no longer significantly differ.

The basic operation of *Step 3* is adjusting the model to fit the data. In this model selection step, 3B makes many local decisions to incrementally adjust the model. It only allows one fluorophore to be either added or removed at a time: either a new fluorophore is added at a random position or a fluorophore in the model is selected for removal. 3B optimizes this spot to search for a new position and then decides whether to keep it in the model. After a series of such decisions have been made, 3B re-optimizes the entire model (*Step 2*) and then repeats the model selection step (*Step 3*).

During the analysis, 3B selects a random position as an initial position of the fluorophore when adding a new fluorophore. 3B considers that the position prior is uniform in all image areas. Intuitively, the distribution of fluorophores is uneven, and the fluorescence intensity at a given position positively correlates with the probability of observing a fluorophore at this position. If the presumed initial position of the fluorophore is far away from the real biological structure, finding the correct position of the fluorophore is difficult. Thus, re-optimizing this position is a waste of time and leads to inaccurate results.

### 3B method based on fluorescence intensity distribution (FID3B)

To improve the reconstruction results and accelerate the calculation, we propose a Bayesian analysis of Bleaching and Blinking microscopy method based on fluorescence intensity distribution (FID3B). In 3B, each fluorophore transfers among an emitting state, non-emitting state and bleached state according to a Markov process, and each transfer is associated with a transition probability. The transition probabilities, together with the fluorophore’s previous state, are used to determine the state of a fluorophore at a certain time. The fluorescence intensity represents the probability of observing a fluorophore at this position. By combining transition probabilities and fluorescence intensity, we calculated the intensity distribution of fluorophores at each pixel. Instead of choosing randomly, we then selected a position with more confidence for each newly added fluorophore.

The key techniques include two aspects: an intensity distribution calculation to obtain the probability distribution of fluorophores and a modified model selection to add a procedure that selects the initial positions of fluorophores based on the intensity distribution.

### Intensity distribution calculation

**Figure 7:**
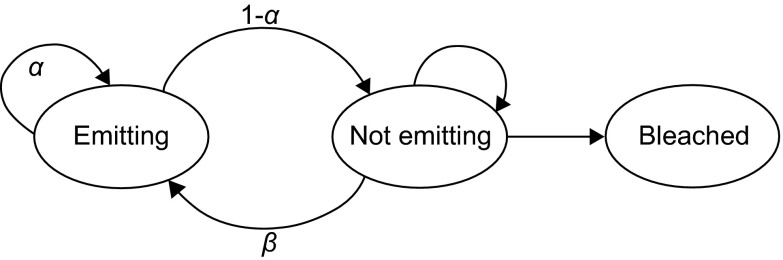
**State transition diagram of fluorophore**

According to the state transition diagram of the fluorophore in 3B, each fluorophore has three possible states, emitting (light), not emitting and bleached, and transfers among the three states. The transition probabilities, i.e., the probability of a fluorophore transferring from emitting to emitting or from not emitting to emitting, are *α* and *β*, respectively, as shown in Fig. 7. We assumed that each fluorophore’s transitions are statistically independent of other fluorophore’s states. This state transition characteristic was used to calculate the probability of the fluorophore at each pixel in the images. We then used these probabilities as an intensity distribution to guide the selection of the initial positions of fluorophores.

Figure 7 shows that two situations cause a fluorophore to emit light: transferring from emitting to emitting and transferring from not emitting to emitting. For convenience, we only considered two states: emitting and not emitting. Like deconSTORM (Mukamel et al., 2012), we assigned an exponential prior distribution to parameter, *γ*_*k*_(*x*), which represents the estimated intensity at location *x* in frame *k*. *γ*_*k*_(*x*) is based on a weighted average of the fluorescence intensity at location *x* in all *K* frames. The weight increases as the distance to the current frame *k* decreases.

2 $$\gamma_{k} (x) =\mathop \sum \limits_{t = 1}^{K} \left( {\frac{{\alpha^{{| {k - t} |}} }}{\tau} + \frac{\beta}{K}} \right)i_{t} (x)$$

where $\tau = \sum\limits_{t = 1}^{K} {\alpha^{|k - t|} }$ is the normalization factor. *K* is the total number of image frames. *i*_*k*_(*x*) represents the fluorescence intensity at location *x* in frame *k*. The first term in Eq. 2 indicates that the observation of image intensity at a particular location in one image frame is generated by a series of image frames. This process calculates a weighted average based on all of image frames, and the current frame is assigned the maximum weight. The weight decreases as the distance from the current frame increases, which decays exponentially. The second term in Eq. 2 indicates that an emitting state detected in any image frame is due to a transition from not emitting to emitting in any earlier or later image frame with probability*β*. We considered the average impact of re-emitting, $\frac{{\sum\nolimits_{t = 1}^{K} {\beta \times i_{t} \left( x \right)} }}{K}$, and combined the first and second term to obtain the estimated intensity, *γ*_*k*_(*x*), at each location in each image frame.

The total intensity at a certain location *x*, *γ*(*x*), is then obtained by adding the estimated intensity, *γ*_*k*_(*x*), in all *K* frames as shown in equation 3.

3 $$\gamma \left( x \right) = \mathop \sum \limits_{k = 1}^{K} \gamma_{k} \left( x \right)$$

Finally, *γ*(*x*) is normalized to obtain probability map of the image at a certain location *x*, *P*(*x*) (Eq. 4). We utilized this probability map as the intensity distribution of fluorophores to evaluate the selection of initial positions. The intensity distribution interval ranges from 0 to 1. A large value represents a high probability of the selected positions of fluorophores.

4 $$P(x) = \frac{\gamma (x)}{{{ \hbox{max} }_{x} \, \gamma (x)}}$$

### Modified model selection

The model selection in 3B (*Step 3* in subsection “Bayesian analysis of bleaching and blinking (3B) method”) is modified to improve the initial positions of fluorophores. When adding a new fluorophore, we used the intensity distribution to select a more reliable initial position. The modified procedures are summarized as follows:

- ***Step 1.*** Select the initial spot positions for a model.
- ***Step 2.*** Optimize the entire model: re-optimizing each fluorophore in turn to obtain a new position in the model.
- ***Step 3.*** Model selection: incrementally adjusting the model to fit the data, one fluorophore at a time. The operation details are shown below:
  - Adding a new fluorophore at a random position (*Op_a*).
  - Adding a new fluorophore based on intensity distribution (*Op_b*).
  - Randomly selecting a fluorophore in the model for removal (*Op_c*).

When model selection (*Step 3*) is executed to adjust the model to fit the data, we take one fluorophore under consideration from three operations: adding a new fluorophore at a random position (*Op_a*), adding a new fluorophore based on intensity distribution (*Op_b*) and randomly selecting a fluorophore in the model for removal (*Op_c*). These three operations are randomly selected with a certain probability. If the probability of *Op_a* is high, FID3B tends to select more random positions as new fluorophores. In contrast, if the probability of *Op_b* is high, FID3B tends to select more fluorophores based on intensity distribution. In particular, if the probability of *Op_b* is set to 0, FID3B is converted to the 3B. In our experiment, the probabilities of the three operations, *Op_a*, *Op_b* and *Op_c,* were set to 0.2, 0.5 and 0.3, respectively.

## Electronic supplementary material

Below is the link to the electronic supplementary material.

Supplementary material 1 (PDF 61 kb)

## Abbreviations

3B: Bayesian analysis of the blinking and bleaching
FID3B: Bayesian analysis of Bleaching and Blinking microscopy method based on fluorescence intensity distribution
PSF: point spread function
FWHM: full width at half maximum
CPU: central processing unit
SSIM: structural similarity
HMM: Hidden Markov Model
FHMM: Factorial Hidden Markov Model
MAP: maximum a posteriori
