# Supplementary material for: Bayesian localization microscopy based on intensity distribution of fluorophores
Source: Protein Cell. 2015 Feb 12;6(3):211–20. doi: 10.1007/s13238-015-0133-9 (PMC4348249; doi:10.1007/s13238-015-0133-9)
Supplement: Supplementary file 1 — Supplementary material 1 (PDF 61 kb) [file 13238_2015_133_MOESM1_ESM.pdf]

Supplemental Materials

To validate the capability of FID3B in resolving fine structures, we generated simulated images with concentric rings structures, where the distance between two neighboring rings is 200 nm (Fig. S1a). As for the previous simulated datasets, we generated 200 image frames from the original image. It is difficult to see the concentric rings structures from the superimposed fluorescence data from 200 frames (Fig. S1b). Here, the FWHM of PSF was set to 240 nm. We ran both 3B and FID3B with a fixed number of iterations, 240, and the reconstruction results are shown in Fig. S1c and Fig. S1d. The reconstruction results of both 3B and FID3B are consistent with the original image. Moreover, the reconstruction result of FID3B has better continuity.

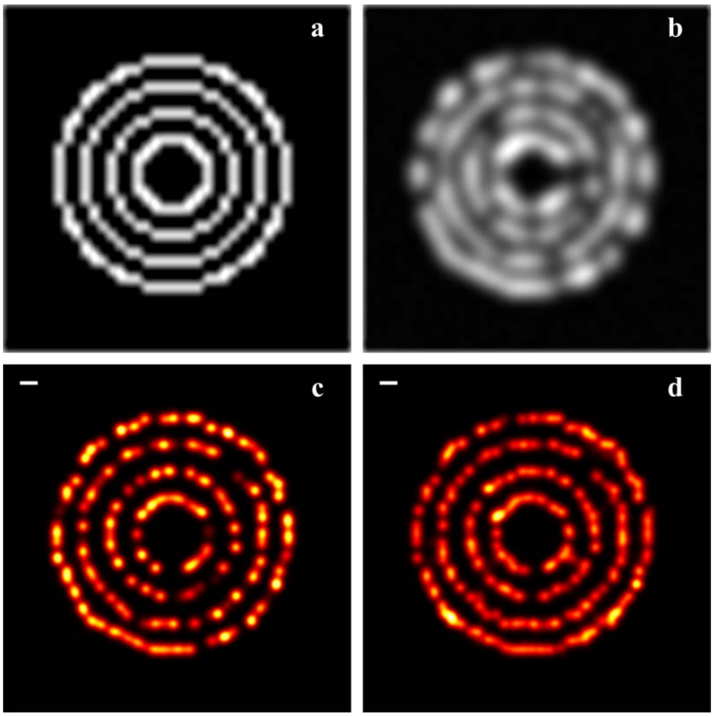

Fig. S1. Comparison of the reconstruction results of 3B and FID3B for the concentric rings dataset. (a) The original image of the concentric rings, where the distance between two neighboring rings is 200 nm. (b) Superimposed fluorescence data from

200 frames representing the diffraction-limited image. (c) Reconstruction result of 3B (240 iterations). (d) Reconstruction result of FID3B (240 iterations). (Scale bar: 200 nm). Note that both 3B and FID3B can address that the structures are quite even in feature distribution. Moreover, the reconstruction result of FID3B has better continuity.
